# The Transcription Factor Mohawk Facilitates Skeletal Muscle Repair via Modulation of the Inflammatory Environment

**DOI:** 10.3390/ijms25095019

**Published:** 2024-05-04

**Authors:** Cherie Alissa Lynch, Sofia A. Acosta, Douglas M. Anderson, Gavin E. Rogers, Jeanne Wilson-Rawls, Alan Rawls

**Affiliations:** 1 School of Life Sciences, Arizona State University, 427 E Tyler Mall, Tempe, AZ 85287, USA; cjlynch1@asu.edu (C.A.L.); saacost6@asu.edu (S.A.A.); doug_anderson@urmc.rochester.edu (D.M.A.); erik.g.rogers@asu.edu (G.E.R.); jeanne.wilson-rawls@asu.edu (J.W.-R.); 2 Molecular and Cellular Biology Graduate Program, Arizona State University, Tempe, AZ 85287, USA

**Keywords:** Mohawk, muscle repair, inflammation, macrophage, cytokine

## Abstract

Efficient repair of skeletal muscle relies upon the precise coordination of cells between the satellite cell niche and innate immune cells that are recruited to the site of injury. The expression of pro-inflammatory cytokines and chemokines such as TNFα, IFNγ, CXCL1, and CCL2, by muscle and tissue resident immune cells recruits neutrophils and M1 macrophages to the injury and activates satellite cells. These signal cascades lead to highly integrated temporal and spatial control of muscle repair. Despite the therapeutic potential of these factors for improving tissue regeneration after traumatic and chronic injuries, their transcriptional regulation is not well understood. The transcription factor Mohawk (*Mkx*) functions as a repressor of myogenic differentiation and regulates fiber type specification. Embryonically, *Mkx* is expressed in all progenitor cells of the musculoskeletal system and is expressed in human and mouse myeloid lineage cells. An analysis of mice deficient for *Mkx* revealed a delay in postnatal muscle repair characterized by impaired clearance of necrotic fibers and smaller newly regenerated fibers. Further, there was a delay in the expression of inflammatory signals such as *Ccl2*, *Ifnγ*, and *Tgfß.* This was coupled with impaired recruitment of pro-inflammatory macrophages to the site of muscle damage. These studies demonstrate that *Mkx* plays a critical role in adult skeletal muscle repair that is mediated through the initial activation of the inflammatory response.

## 1. Introduction

Skeletal muscle possesses an intrinsic ability to repair itself in response to traumatic or chronic injury. Repair is dependent on signaling crosstalk between the myogenic satellite cells and the innate immune system [1,2]. Damage to the myofiber leads to the release of chemokines and T helper type 1 (Th1) cytokines that both activate and expand satellite cells, in addition to initiating a pro-inflammatory response that directs myolysis. This is followed by a transition to the release of T helper type 2 (Th2) cytokines that promote differentiation and fusion of satellite cells, as well as activation of an anti-inflammatory response [3]. Disruption of these signals promotes fibrotic scarring and reduced muscle function. Understanding the regulation of Th1 and Th2 cytokine expression during skeletal muscle injury is essential for promoting robust tissue repair and mitigating fibrosis.

Damage to the myofiber initiates a rapid recruitment of neutrophils, mediated by the release of the chemoattractant C-X-C Motif Chemokine Ligand 1 (CXCL1) from resident macrophages. Release of interleukin-1 (IL-1) and interleukin-8 (IL-8) from neutrophils and C-C Motif Chemokine Ligand 2 (CCL2) from skeletal muscle resident macrophages recruits circulating monocytes to the site of damage [4,5]. Monocytes differentiate into a pro-inflammatory macrophage subtype, termed M1, which are characterized by the expression of F4/80, Ly6C, and CD11b [6]. M1 macrophages clear necrotic fibers by phagocytosis and promote satellite cell activation and proliferation through the release of reactive oxygen species and inflammatory cytokines, such as interferon-γ (IFNγ) and tumor necrosis factor-α (TNFα) [7,8,9]. M1 macrophages reach peak numbers 1–2 days following injury, before progressively transitioning to an anti-inflammatory and pro-regenerative M2 macrophage phenotype. M2 macrophages, reaching peak numbers 3–5 days post-injury, express the mannose receptor CD206 and secrete cytokines, such as Interleukin-10 (IL-10) and transforming growth factor β1 (TGFβ), to aid in the resolution of inflammation and differentiation of satellite cells [1,10]. We recently demonstrated that lipopolysaccharide (LPS) stimulation of satellite cells in culture resulted in their expression of pro-inflammatory chemokines, cytokines, and interferon (IFN) signaling pathway members [11]. Further, in response to cardiotoxin (CTX)-induced damage, both the muscle and satellite cells at the site of injury expressed *Ccl2* and *Ccl5,* indicating that this also occurs in vivo [11]. These data indicate that the muscle may also participate in inflammation and recruitment of innate immune cells in response to damage.

The timely initiation of inflammation and its subsequent cessation during muscle regeneration is critical for efficient repair. Reduced infiltration of M1 macrophages due to genetic ablation of *Ccl2,* or its receptor *Ccr2,* resulted in diminished growth of newly repaired fibers after injury [5,12]. Similarly, depletion of F4/80^+^ macrophages at the transition point from a pro-inflammatory to a pro-regenerative environment resulted in reduced satellite cell differentiation and impaired muscle regeneration [13]. Alternatively, chronic inflammation associated with muscular dystrophies leads to accumulation of fibrotic scarring and severe loss of muscle function. Continuous expression of pro-inflammatory cytokines exacerbates muscle damage by halting the transition of M1 macrophages to the M2 subtype and inhibiting the apoptosis of fibro-adipogenic progenitors (FAPs). Under chronic inflammatory conditions, TGFβ causes FAPs to differentiate into fibroblasts, leading to increased deposition of extracellular matrix (ECM) [14,15]. Understanding the regulation of the highly coordinated and intricate signaling network that exists between the satellite cell niche and the innate immune response is critical for the development of therapeutics, as much is still unknown about the signals that regulate efficient regeneration.

Mohawk (*Mkx*), a member of the TALE superclass of atypical homeobox genes, is a transcriptional repressor associated with the Sin3A/HDAC co-repressor complex [16,17]. MKX binds a bipartite DNA recognition sequence consisting of a highly conserved inverted repeat (ATGTT-N_0–25_-AACAT) [18]. In developing mouse embryos, *Mkx* is expressed in the dorsomedial and ventrolateral lips of the dermomyotome of maturing somites [16]. *Mkx* also is expressed in the ureteric buds of the metanephric kidney, sex chords of the testis, developing inner ear, and in the progenitors of tendons and cartilage of the limbs and tail [16,19,20]. MKX is a regulator of tendon differentiation; a deficiency of MKX embryonically resulted in hypoplastic tendons due to a significant reduction of extracellular matrix components leading to decreased tendon size, growth, and abnormal sheath development [16,17,19,20,21,22]. MKX has been shown to regulate the expression of tendon-specific genes including decorin *(Dcn*), tenomodulin (*Tnmd*), collagen 1a1 (*Col1a1*), and collagen 2a1 (*Col2a1*) [23,24,25]. Human bone marrow-derived mesenchymal stem cells transduced with *Mkx* underwent tenogenesis both in vivo and in vitro [25]. *Mkx* has also been shown to be important for the development, maintenance, and regeneration of the annulus fibrosus [26].

*Mkx*-deficient embryos demonstrated no overt skeletal muscle phenotype [19,21], but in cultured 10T1/2 mesenchymal stem cells, expression of *Mkx* inhibited myogenic differentiation [17]. MKX also inhibited the expression of *Myh7*, indicating its role in regulating fiber type specificity [18]. Similarly, in tendon-derived cells (TDCs) lacking *Mkx*, the differentiation of chondrogenic and osteogenic cells was markedly enhanced, while over-expression of *Mkx* lead to decreased differentiation of chondrogenic, osteogenic, and adipogenic cells [19]. MKX inhibited differentiation of tendon progenitor cells (TSPCs) into myofibroblasts, indicating a role in limiting fibrosis during tendon healing [27].

While *Mkx* is expressed in musculoskeletal progenitor cells of muscle, tendon, cartilage, and bone [16], examination of several mouse and human datasets demonstrated that it is also expressed in basophils, eosinophils, and monocytes/macrophages [28]. This expression pattern and the role of *Mkx* in regulating muscle differentiation suggested that *Mkx* may play a critical role in muscle repair. We wanted to examine the potential roles of *Mkx* in both myogenic and innate immune cells during skeletal muscle repair in response to injury. In these studies, an evaluation of *Mkx*-deficient mice (*Mkx^−/−^*) demonstrated delayed muscle regeneration and aberrant macrophage recruitment in response to acute injury. Further, a deficiency of MKX delayed the onset of the pro-inflammatory response post-damage in vivo, while increasing the rate of muscle differentiation in vitro. These data revealed a regulatory role for *Mkx* in the timing of skeletal muscle regeneration.

## 2. Results

### 2.1. Impaired Muscle Regeneration in Mkx^−/−^ Mice

Previous studies demonstrated that *Mkx*-deficient mice have hypoplastic tendons, but no differences were observed in the skeletal muscle, indicating that *Mkx* is not required for embryonic muscle development [18,19,21]. Since *Mkx* is expressed in muscle progenitor cells [14], we determined whether MKX was involved in the regulation of postnatal muscle regeneration. These experiments were performed using the well-characterized CTX-induced injury model in wild type and *Mkx^−/−^* mice. This acute muscle injury is well studied and induces an immediate inflammatory response, followed by activation of satellite cells, differentiation of myotubes, and myofiber maturation, over a 21-day period [1].

**Figure 1:**
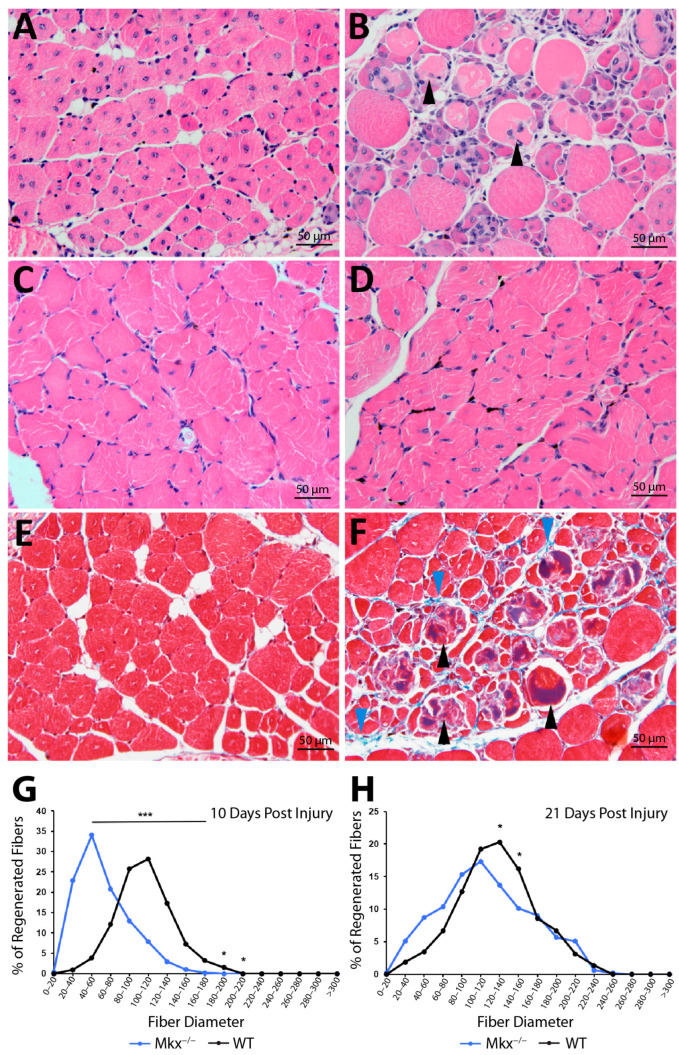
Delayed muscle regeneration in *Mkx^−/−^* mice following Cardiotoxin-induced damage. (**A**) Hematoxylin and eosin stained transverse sections at 10 days post injury in WT and (**B**) *Mkx^−/−^* quadriceps. Black arrowheads denote necrotic fibers. (**C**) Hematoxylin and eosin stained transverse sections at 21 days post-injury in WT and (**D**) *Mkx^−/−^* quadriceps. (**E**) Masson’s trichrome stained transverse sections at 10 days post-injury of WT and (**F**) *Mkx^−/−^* quadriceps. Black arrowheads denote necrotic fibers and blue arrowheads denote collagen deposition. (**G**) Distribution of fiber diameters in regenerating WT and *Mkx^−/−^* muscle at 10 days post-injury and (**H**) 21 days post injury. Fiber measurements are displayed in pixel units. Data are the mean ± SD of 5 replicates/genotype/timepoint, * *p* ≤ 0.05 and *** *p* ≤ 0.001. Photomicrographs are 10× magnification.

Since MKX may play regulatory roles in the injury responses of both skeletal muscle and innate immune cells, we compared the *quadriceps femoris* (quads) of wild type (WT) and *Mkx^−/−^* mice. Muscles of 3 month old mice were injected with CTX and tissue was harvested and assessed at 10- and 21-days post-injury (DPI). At both timepoints, histological sections of *Mkx^−/−^* and WT quads revealed muscle fibers with centralized nuclei, indicating fiber regeneration (Figure 1A–D). However, at 10 DPI, the *Mkx^−/−^* muscle contained many necrotic muscle fibers that are normally cleared by M1 macrophages in the first 3 DPI (Figure 1B,F). To quantify the robustness of muscle repair, the minimal Feret’s diameter of myofibers with centralized nuclei was measured using ImageJ (version 1.53k) (Figure 1G,H). At 10 DPI, the average diameter of regenerated fibers in *Mkx^−/−^* muscle was 62.29 +/− 11.54, while the average diameter of the regenerated fibers in WT muscle was 106.73 +/− 8.37, *p* < 0.001 (Figure 1G). The WT muscle had significantly larger diameter myofibers than *Mkx^−/−^* muscle. A comparison of *Mkx^−/−^* muscle from 10 and 21 DPI revealed that necrotic fibers were cleared (Figure 1B,D). At 21 DPI, the overall average diameter of myofibers in *Mkx^−/−^* muscle was 115.4 +/− 27.56, whereas WT was 129.23 +/− 17.6, *p* = 0.18 (Figure 1H). While the difference was not significant, the trend of larger diameter fibers in the repairing WT muscle continued, indicating that the *Mkx^−/−^* muscle had not caught up with the WT muscle. These data demonstrate that a lack of *Mkx* results in inefficient clearance of injured fibers, and delayed muscle fiber regeneration.

### 2.2. Mkx Regulation of Muscle Satellite Cell Proliferation and Differentiation

**Figure 2:**
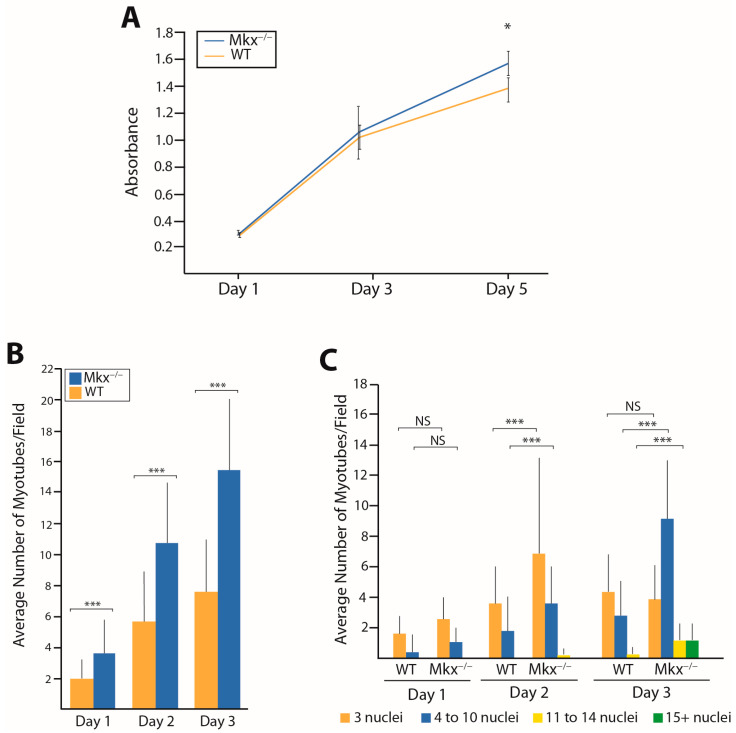
Comparison of proliferation and differentiation of *Mkx^−/−^* and WT satellite cells. (**A**) MTT assays were done using 6 × 10^4^ cells/well on Cultrex-coated 24-well plates in growth medium. Data are average absorbance at 570 nm +/− s.d. of three biological replicates analyzed in triplicate. Significance was assessed by ANOVA, * *p* < 0.05. (**B**) Differentiation assays—1 × 10^5^ satellite cells were seeded into Cultrex-coated 35 mm plates. At 24 h differentiation medium was added, cells were fixed at days 1–3, and nuclei stained with DAPI. Myotubes with a minimum of 3 nuclei were counted in 10 random 10× magnification fields/plate. Data are average myotubes/field +/− s.d. of 5 experiments. (**C**) Size distribution of myotubes based on number of nuclei. Data were analyzed by ANOVA, *** *p* < 0.001, NS = not significant.

Since muscle progenitor cells express *Mkx* embryonically and overexpression of this gene inhibited the differentiation of muscle and tendon progenitors [17,19,21,22,27], it was possible that the delayed regeneration response was due to defects intrinsic to the muscle satellite cells (MuSCs) (Figure 2). Proliferation was assessed by MTT assay using MuSCs freshly isolated from the quads of *Mkx^−/−^* and WT mice. MuSCs were seeded at 6 × 10^4^ cells/well in Cultrex-coated 24-well plates in growth medium. Absorbance was assessed at days 1, 3, and 5. The *Mkx^−/−^* MuSCs grew at the same rate at WT MuSCs, indicating that the delay in muscle repair was not due to a proliferation deficit in the muscle stem cells (Figure 2A). We next examined the ability of *Mkx*-deficient satellite cells to differentiate. Freshly isolated *Mkx^−/−^* and WT satellite cells were plated on Cultrex-coated plates and incubated in differentiation medium. Myotubes with a minimum of 3 nuclei were counted in random 10X fields over a 3-day period (Figure 2B). *Mkx^−/−^* satellite cells demonstrated a significantly higher number of myotubes than WT at each timepoint (Figure 2B). When examining the size distribution of myotubes based on the number of nuclei, *Mkx^−/−^* plates had significantly more myotubes with 4–10 nuclei and only *Mkx*-deficient cells formed larger myotubes with 11–14 nuclei at day 2 (Figure 2C). By day 3, *Mkx^−/−^* cultures had significantly more myotubes with 11–14 and greater than 15 nuclei (Figure 2C). These data indicate that the *Mkx^−/−^* satellite cells differentiate faster into larger myotubes, consistent with previous studies demonstrating that MKX limits differentiation [17].

### 2.3. Inflammatory Cell Recruitment to the Site of Muscle Injury

**Figure 3:**
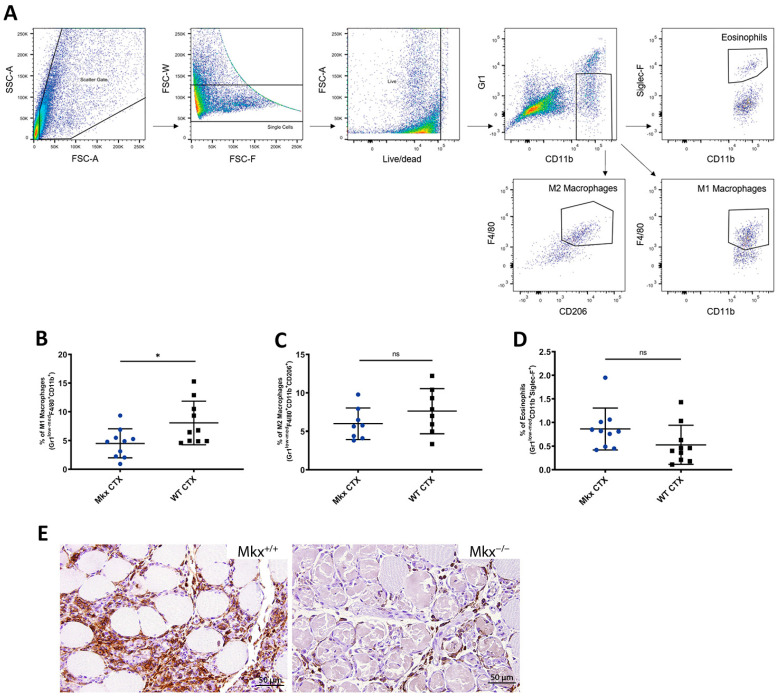
Deficit of M1 macrophages in *Mkx^−/−^* muscle after injury. M1 macrophages were significantly reduced in *Mkx^−/−^* muscle post-cardiotoxin injury. (**A**) Gating strategy for FACS analysis of macrophage and eosinophil populations in muscle. (**B**) Quantification of M1 macrophages, (**C**) M2 macrophages, and (**D**) eosinophils at 2 days post-injury in *Mkx^−/−^* and WT muscle. Data are the mean ± SD of 5 experiments, each experiment done with 5 mice, * *p* ≤ 0.05, ns—not significant. (**E**) Representative 10× magnification photomicrographs of anti-F4/80 I HC staining of injured WT and *Mkx^−/−^* muscle at 3 days post-injury.

The persistence of necrotic fibers at 10 DPI that was observed in *Mkx^−/−^* muscle has been reported in mice deficient for the macrophage chemoattractant CCL2 [5]. This suggested that an impairment of M1 macrophage infiltration could be responsible for the delay in clearance of necrotic myofibers in *Mkx^−/−^* muscle. Consistently, using the Haemosphere databases [28] to examine gene expression of *Mkx* in hematopoietic lineages, we found, in both mouse and human cells, that this gene was expressed in eosinophils, neutrophils, monocytes, and macrophages. Thus, a lack of *Mkx* could be affecting the function of the immune effector cells during muscle regeneration. To examine this, M1 macrophages were quantified via flow cytometric analysis (FACS) in CTX-injured and contralateral control quadriceps muscles at 2 DPI, when the pro-inflammatory response is at its peak and there should be maximal macrophage infiltration. Cells were stained with fluorescently conjugated F4/80, CD11b, and Gr1 antibodies to identify M1 macrophages (see gating strategy in Figure 3A). In CTX-injured *Mkx^−/−^* muscle, there were significantly fewer M1 macrophages (Gr1^low-med^, F4/80^+^, CD11b^+^) in comparison to WT injured muscle (Figure 3B). The reduced number of Gr1^low-med^, F4/80^+^, and CD11b^+^ cells at 2 DPI confirmed a significant decrease in the number of M1 macrophages recruited to the site of injury in *Mkx^−/−^* muscle during the pro-inflammatory phase.

To rule out the possibility of premature polarization, the M2 macrophage population was examined at 2 DPI. Eosinophils, which rapidly infiltrate damaged muscle [29], were also examined. FACS demonstrated no significant differences in the population of M2 macrophages or eosinophils (Figure 3C,D). Importantly, the FACS showed that the uninjured *Mkx^−/−^* and WT contralateral muscles demonstrated no significant difference in the number of M1 or M2 macrophages, or eosinophils, indicating there is not a decrease in the number of immune effector cells in homeostatic muscle. Immunohistochemical staining for a pan-macrophage marker, F4/80, in injured muscle at 2 DPI confirmed a decrease in inflammatory cell recruitment to the site of damage (Figure 3E).

### 2.4. Compromised Innate Immune Response in Mkx^−/−^ Mice

**Figure 4:**
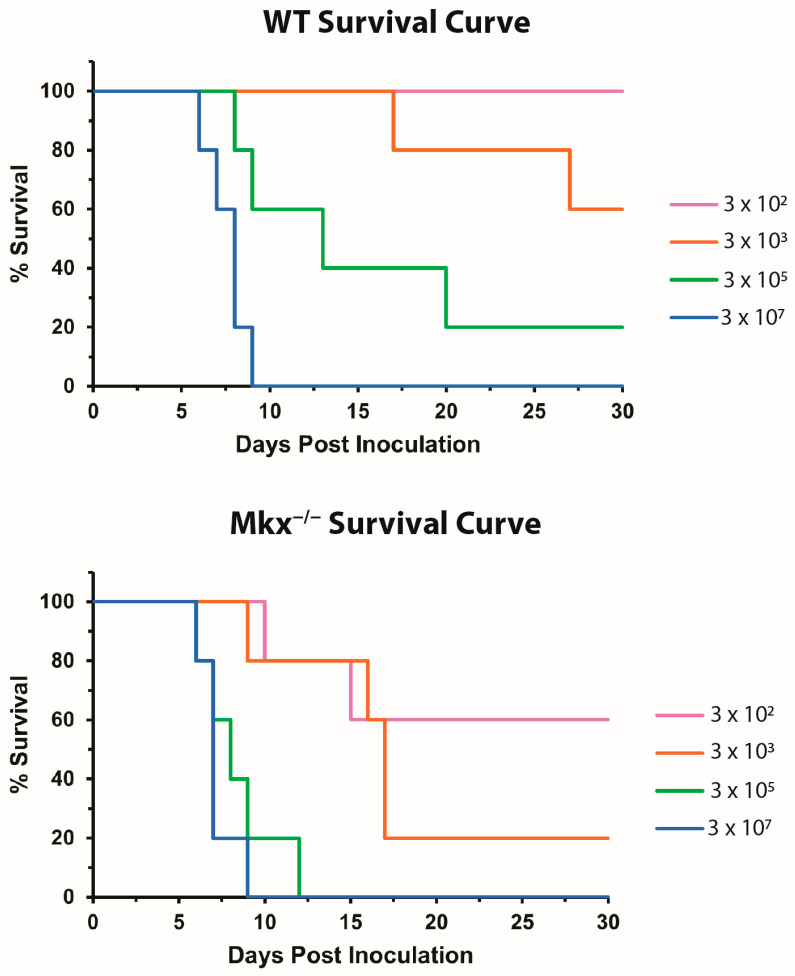
*Mkx^−/−^* mice have increased susceptibility to bacterial infection. *Mkx^−/−^* and WT mice, n = 5 mice/dose/genotype, were inoculated with 10^2^, 10^3^, 10^5^, and 10^7^ CFU of *S. enterica* and Kaplan–Meier survival curves were generated. *Mkx^−/−^* mice demonstrated increased susceptibility to lower doses of bacteria.

M1 macrophages are a central component of the innate immune response for both tissue repair and infection [30]. To assess whether a lack of MKX can impair systemic M1 macrophage recruitment during infection, mice were challenged with the bacteria *Salmonella enterica* serovar Typhimurium. *Mkx^−/−^* and WT mice were inoculated with four serial doses of *S. enterica* and the virulence was assessed by determination of the bacterial dose causing lethality in 50% of the exposed population (LD_50_). In comparing the Kaplan–Meier survival curves, it is clear that *Mkx^−/−^* mice were more susceptible to lower doses of *S. enterica*, than WT mice over a 17-day incubation period (Figure 4). At a dose of 10^2^ colony forming units (CFU), 100% of WT mice were able to resolve the infection, while 40% of *Mkx^−/−^* mice succumbed. Overall, the LD_50_ for *Mkx^−/−^* mice was 5.3 × 10^2^ CFU, in comparison with an LD_50_ of 3.7 × 10^4^ CFU for WT mice. This is a 70-fold increase in susceptibility of *Mkx^−/−^* mice to *S. enterica* and suggests that *Mkx* is necessary for M1 macrophage recruitment in the activation of pro-inflammatory immune responses.

### 2.5. Polarization and Proliferation of Mkx^−/−^ Bone Marrow-Derived Macrophages In Vitro

**Figure 5:**
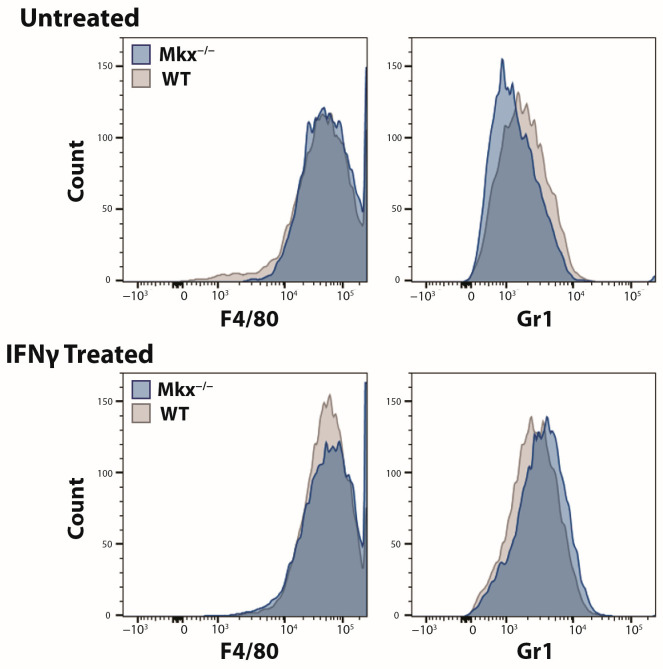
Proficient polarization of *Mkx^−/−^* bone marrow-derived macrophages. MKX-deficient cells demonstrate no lack of polarization to the Gr1^+^ M1 subtype. Representative FACS analysis of F4/80 and Gr1 surface expression from WT and *Mkx^−/−^* bone marrow-derived cells +/− 10 ng/mL IFNγ. Grey histogram peaks denote WT bone marrow-derived cells and blue histogram peaks denote *Mkx^−/−^* bone marrow-derived cells, n = 3.

In response to injury or infection, macrophages polarize into the M1 pro-inflammatory subtype, and these cells secrete proinflammatory cytokines. We next addressed whether the diminished immune response was an intrinsic defect of proliferation and/or polarization of *Mkx^−/−^* macrophages. Bone marrow-derived cells (BMDCs) were isolated from the femurs of WT and *Mkx^−/−^* mice and tested for their ability to polarize to M1 macrophages and proliferate in culture. BMDCs were treated with IFNγ (10 ng/mL) [31] to induce polarization to the M1 pro-inflammatory subtype. After 3 days, IFNγ-treated and untreated BMDCs were analyzed using FACS to detect the macrophage differentiation marker, F4/80, and the M1 macrophage-specific surface marker, Gr1. In both treated and control cells, *Mkx^−/−^* and WT peaks overlap and the cells all express F4/80 (Figure 5). IFNγ treatment of WT and *Mkx^−/−^* BMDCs caused a shift from Gr1^−^ to Gr1^+^ cells in *Mkx^−/−^* and WT cells as evidenced by the rightward shift of the peaks, indicating these cells were polarized towards the M1 subtype (Figure 5). These results indicate that *Mkx* does not participate in the regulation of BMDC polarization to the pro-inflammatory phenotype.

**Figure 6:**
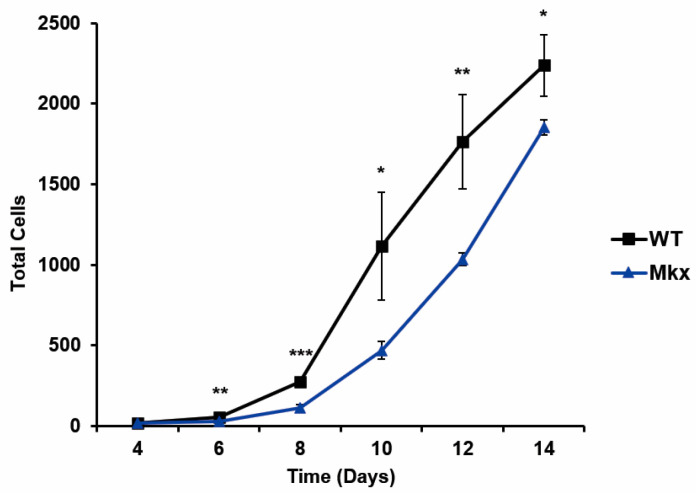
Delayed proliferation of *Mkx^−/−^* bone marrow-derived macrophages. Proliferation of WT and *Mkx^−/−^* bone marrow-derived cells over 14 days in culture was determined using Trypan blue exclusion counting. Data are mean ± SD from 3 experiments done in triplicate, * *p* ≤ 0.05, ** *p* ≤ 0.01, *** *p* ≤ 0.001.

It was also possible that the diminished response to injury in the *Mkx^−/−^* muscle was due to a reduced proliferation capacity of macrophages. Therefore, proliferation was examined using the same culture conditions, without IFNγ, with manual cell counting done at day 4 and continuing every 2 days over a two-week period. For each timepoint, independent dishes of cells were removed, stained with Trypan blue, and live cells were counted. *Mkx^−/−^* macrophages exhibited a significant reduction in the increase in cell number when compared to WT cells (Figure 6), suggesting a role for *Mkx* in regulating macrophage proliferation.

### 2.6. Altered Expression of Cytokines in Mkx^−/−^ Skeletal Muscle following Injury

Cytokines and chemokines essential for regulating the immune response to injury are expressed by both resident macrophages and skeletal muscle [4,5]. We next determined if a lack of *Mkx* affected the transcription of *Ccl2*, *Tnfα*, and *Ifnγ*. Total RNA was isolated from *Mkx^−/−^* and WT quadriceps tissue after CTX-induced injury at 1, 2, 3, and 5 DPI. Muscle from the contralateral uninjured quadriceps was used as a control. Quantitative RT-PCR was performed using gene-specific primers to compare transcription between *Mkx^−/−^* and WT mice.

**Figure 7:**
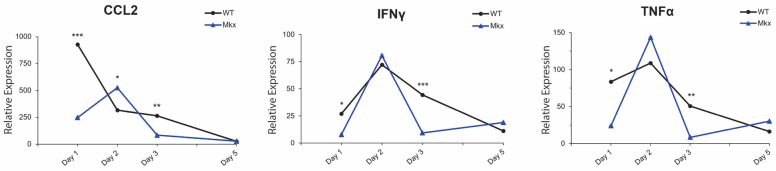
Altered inflammatory response in *Mkx^−/−^* muscle following cardiotoxin-induced injury. *Mkx*-deficient muscle demonstrated reduced expression of the pro-inflammatory genes, *Ccl2*, *Ifnγ,* and *Tnfα*. Total muscle RNA was isolated from *Mkx^−/−^* and WT cardiotoxin-injured quads and transcription levels were analyzed by RT-qPCR using ΔΔCt analysis. All samples (n = 5 at day 1, n = 9 at day 2, n = 4 at day 3, n = 3 at day 5) were normalized to WT uninjured contralateral muscle. Data are the mean ± SD of each timepoint, * *p* ≤ 0.05, ** *p* ≤ 0.01, *** *p* ≤ 0.001.

In response to CTX injury, as compared to WT muscle, the transcription level of *Ccl2,* a pro-inflammatory chemotactic factor for monocytes, was 3 times lower in *Mkx^−/−^* quads. Its transcription peaked at 2 DPI and decreased sharply by 3 DPI (Figure 7). *Tnfα*, another pro-inflammatory cytokine, showed a rapid rise in transcription to a peak at 2 DPI followed by a rapid decline. *Ifn*γ, which was recently shown to be expressed by both muscle fibers and satellite cells [11], mirrored the pattern of transcription of *Tnfα* (Figure 7). *Ifnγ* and *Tnfα* are both secreted by M1 macrophages and exhibited similar peak expression to that detected in WT muscle, but the response was sharply attenuated by 3 DPI (Figure 7). The downregulation of these inflammatory markers point to a possible role for *Mkx* in mounting a robust pro-inflammatory immune response during the early stages of muscle repair. Collectively, these data demonstrated that in the absence of MKX, the onset of the pro-inflammatory response was delayed in response to muscle damage. This, and the reduced proliferative rate of *Mkx^−/−^* macrophages are potential mechanisms for the observed aberrant macrophage recruitment in these mice.

## 3. Discussion

*Mkx* is a member of the TALE superclass of atypical homeobox genes that plays an important role in the development of tendons and ligaments [19,21,26]. The present studies demonstrate a novel role for *Mkx* during skeletal muscle regeneration in response to acute injury. Injured *Mkx^−/−^* muscle exhibited a delay in the removal of necrotic muscle fibers, reduced recruitment of M1 macrophages to the site of the injury, and decreased expression of pro- and anti-inflammatory cytokines. Additionally, *Mkx^−/−^* mice exhibited increased susceptibility to infection by *S. enterica*, suggesting a link between the suppression of the pro-inflammatory immune response and the delay in muscle repair.

Following an acute injury in muscle, CCL2 is released by resident macrophages to recruit circulating monocytes to the site of damage [4,5]. M1 macrophages, which are derived from monocyte precursors, are essential for the clearance of necrotic fibers [7,8]. Genetic ablation of *Ccl2*, or its receptor *Ccr2*, impaired macrophage infiltration and phagocytosis at the site of induced muscle injury [5]. Interestingly, the injury phenotype of the muscle in *Ccl2^−/−^* and *Ccr2^−/−^* mice resembles that of *Mkx^−/−^* skeletal muscle, in which there was delayed clearance of necrotic fibers and smaller regenerating myofibers. Consistently, the expression of *Ccl2* was delayed and decreased in damaged *Mkx^−/−^* muscle. A similar regulatory relationship was observed between *Mkx*, *Tnfα*, and *Ifnγ*. These cytokines, secreted by resident immune cells in skeletal muscle, are necessary to elicit a robust pro-inflammatory response post-injury [7,8] and synergize to activate M1 macrophages [14]. IFNγ expression increases simultaneously with the influx of neutrophils, macrophages, and *MyoD*^+^ satellite cells in damaged muscle. *Ifnγ^−/−^* mice exhibit diminished muscle repair, impaired macrophage function, and increased fibrosis [8]. *Mkx^−/−^* mice displayed delayed, less efficient muscle repair (Figure 1). This is consistent with the finding that *Mkx* participates in regulating the timing of pro-inflammatory cytokine expression in response to injury (Figure 7).

In response to injury, *Mkx^−/−^* muscle demonstrated significantly smaller myofibers and this persisted at 21 DPI (Figure 1). Injured muscle lacking MKX also demonstrated decreased expression of pro-inflammatory cytokines as compared to WT (Figure 7). When isolated, *Mkx*-deficient and WT satellite cells had similar growth curves (Figure 2), indicating that there is no intrinsic proliferation defect. When differentiated in vitro, *Mkx^−/−^* satellite cells formed more multinucleated myotubes earlier than WT cells (Figure 2). These data indicate that in the absence of *Mkx* the satellite cells may differentiate prematurely reducing the activated cell population. *Mkx* is also expressed in the cells of the innate immune response [28], raising the possibility that the gene is directly regulating recruitment of M1 macrophage to the site of injury. Our studies with BMDCs in culture predict that Mkx plays a cell autonomous role in regulating macrophage proliferation but not polarization to the pro-inflammatory M1 macrophage type. A failure of the macrophage population to expand would contribute to reduced recruitment at the site of injury.

The innate immune response associated with sterile tissue repair, like that observed in skeletal muscle, is shared with bacterial infection [30]. To determine whether the reduced pro-inflammatory response is indicative of a broader immune deficit, *Mkx^−/−^* mice were infected with *S. enterica*, a gram-negative bacterium that invades the mucosa of the gastrointestinal tract and causes an acute inflammatory reaction. In these studies, *Mkx^−/−^* mice were found to have a 70-fold increase in susceptibility to *S. enterica*. This is consistent with a systemic role for *Mkx* in the induction of pro-inflammatory M1 macrophages. However, *S. enterica* infection can occur through multiple pathways, including via M cell mediated transcytosis at the Peyer’s patches [32]. Thus, it is also possible that MKX plays an additional role in other cell types (e.g., M cells) that were not investigated here.

The precise cell types regulated by *Mkx* during the pro-inflammatory response have yet to be determined. The Haemosphere databases indicated that among the many innate immune cell types, *Mkx* is expressed at high levels in eosinophils [28]. Interestingly, Heredia et al. [33] demonstrated that eosinophils are rapidly recruited to the site of muscle damage, where they activate the pro-regenerative function of FAPs via secretion of IL-4. Further, mice that are eosinophil deficient fail to regenerate skeletal muscle post-injury, indicating that these cells and Mkx may have an important regulatory role in regeneration [33]. Further research into the precise function of *Mkx* in these and other innate immune cells could provide valuable insight for the development of potential therapeutic approaches aimed at regulating inflammation and regeneration.

## 4. Materials and Methods

### 4.1. Mice and Genotyping

*Mkx^−/−^* mice on a 129C57BL6/J mixed background have been previously reported (16). WT 129C57BL/6J mice were purchased from The Jackson Laboratories (Bar Harbor, ME, USA). All animals used were bred and maintained in the vivarium at Arizona State University (ASU) on a 10 h light:14 h darkness cycle with ad libitum access to food and water. ASU is accredited by AALAC and all animal procedures were carried out in compliance with the ASU institutional animal care and use committee under an approved research protocol. *Mkx^−/−^* mice were genotyped and maintained as previously described [16].

### 4.2. Muscle Injury

Controlled injury of the right quadriceps of *Mkx^−/−^* mice and WT age-matched control mice at 3 months of age was induced by injection of 50 μL of cardiotoxin (10 μM) (Cat# L8102-1MG, Latoxan, Westbury, NY, USA) [34]. In some cases, 24 h prior to tissue harvesting, an i.p. injection of 1% Evans blue dye (Cat# E2129, Sigma-Aldrich, St. Louis, MO, USA) was administered to allow for visualization of damaged cellular membranes [35]. Mice were sacrificed at designated time points and tissue was harvested for analysis. The uninjured quadriceps muscle on the contralateral leg was also harvested for use as controls.

### 4.3. Histology

Muscles were dissected from euthanized mice and fixed overnight at 4 °C in 4% paraformaldehyde. The tissues were then washed in phosphate buffered saline (PBS), dehydrated through serial ethanol dilutions, and embedded in paraffin (Cat#39503002, McCormick Scientific, New York, NY, USA). Tissues were sectioned at 5 μm, stained with hematoxylin and eosin (H&E) or Masson’s trichrome and imaged using CellSens software (version 1.18) and an Olympus BX50 microscope (Breinigsville, PA, USA). The minimal Feret’s diameter of muscle fibers containing centralized nuclei was manually measured on transverse H&E stained sections using ImageJ software (version 1.53k).

### 4.4. Cell Culture and Satellite Cell Isolation

Muscle progenitor cells were isolated from 12 week old WT or *Mkx^−/−^* mice, as previously described [31]. Briefly, hind limb quadriceps femoris muscles were excised, trimmed of fat and connective tissue, and finely minced. The muscle tissue was digested with 1.25 mg of protease XIV (Cat# P5147, Sigma-Aldrich) for 1 h at 37 °C. The cell suspension was filtered, differentially centrifuged, and pre-plated in DMEM (Corning, Corning, NY, USA), containing 2% donor horse serum (HS) (Atlanta Biologicals, Flowery Branch, GA, USA), and 100 μg/mL Primocin (Cat# ant-pm-05, InvivoGen, San Diego, CA, USA). Satellite cells were grown in a humidified chamber at 37 °C with 5% CO_2_, on Cultrex (Cat# 3432-010-01, R&D Systems, Minneapolis, MN, USA) coated tissue culture plates in growth medium; Hams F-10 (Cat# 11550043, Gibco, Grand Island, NY, USA), 20% FBS (R&D Systems), 10 ng/mL bFGF (Cat# 354060, BD Biosciences, Bedford, MA, USA) and 100 μg/mL Primocin (InvivoGen, San Diego, CA, USA).

### 4.5. MTT Proliferation Assays

Satellite cells were passaged once, then seeded at 6 × 10^4^ cells per well in Cultrex-coated 24-well plates (R&D Systems) in Ham’s F10 (Gibco) growth medium supplemented with 20% FBS (R&D Systems), bFGF (BD Biosciences), and Primocin (InvivoGen). On days 1, 2, 3, and 5 post-plating, cells were incubated with 12 mM MTT (3-(4,5-dimethylthiazol-2-yl)-2,5-diphenyltetrazolium bromide) (Cat# 6494, Molecular Probes, Eugene, OR, USA) in phenol red-free Ham’s F10 with 20% FBS (R&D Systems) for 4 h at 37 °C. SDS-HCl was added, cells were incubated an additional 4 h at 37 °C, mixed thoroughly and 150 μL/well were transferred into a 96-well plate and absorbance was read at 570 nm. All data are the average +/− standard deviation of three technical replicates from 3 biological replicates per time point.

### 4.6. Satellite Cell Differentiation

WT and *Mkx^−/−^* satellite cells were switched to differentiation medium (DMEM, 2% HS, 100 μg/mL Primocin (Invivogen) 24 h post-plating on 35 mm Cultrex-coated plates. The medium was changed daily. Cells were cultured for 1, 2, or 3 days before fixation and imaging. Cultures were fixed with 4% paraformaldehyde (PFA) (Sigma Aldrich) in 4 °C PBS at RT, washed 3 times in PBS. Nuclei were visualized by DAPI staining (Cat# 62248, ThermoFisher Scientific, Waltham, MA, USA). DAPI was diluted 1:1000 in diH_2_O and incubated for 10 min at RT, rinsed 3 times in PBS and stored at 4 °C until imaging. Myotubes containing a minimum of three nuclei were counted in 10 random 10× magnification fields per plate. All time points were done in triplicate.

### 4.7. Bone Marrow-Derived Macrophage Culture, Proliferation Assay, and Polarization

Bone marrow was harvested from femurs as described previously [36,37]. Bone marrow-derived cells (BMDC) from 5 mice were pooled, plated at 4 × 10^5^ cells per 100 mm petri dish and cultured in RPMI 1640 (VWR International, Radnor, PA, USA) supplemented with M-CSF from L929-conditioned media and heat-inactivated FBS (R&D Systems) [36]. Macrophage differentiation was verified by flow cytometric (FACS) analysis of F4/80^+^ stained cells. For the proliferation assay, 1 × 10^5^ cells were plated per triplicate well per experimental time point, in a 6-well non-treated plate. A partial media change was performed every 2 days to maintain the concentration of M-CSF. Adherent cells were collected in PBS, stained with Trypan blue (Cat# 15250061, Sigma-Aldrich), and counted using a hemocytometer and an Olympus CK40 microscope (Breinigsville, PA, USA). To induce polarization, BMDCs were plated at 36,000 cells/cm^2^, in triplicate per condition, in a non-treated 24-well plate and stimulated with 10 ng/mL IFNγ (Cat# 285-IF-100/CF, R&D Systems) 6 h after plating [29]. Cells were harvested 3 days later, washed, and stained with F4/80 and Gr1. Polarization efficacy was assessed via FACS.

### 4.8. mRNA Isolation and Quantitative Real-Time PCR

RNA was extracted from isolated *Mkx^−/−^* and WT cells or whole quadriceps tissue with TRIzol (Life Technologies ThermoFisher, Waltham, MA, USA) according to the manufacturer’s protocol. Reverse transcription of 1 μg of total RNA was performed using Superscript III (Cat#18080093, Invitrogen, Waltham, MA, USA). Real-time quantitative PCR analysis of the cDNA was performed using qPCR MasterMix Plus without UNG (Cat#18080093, Eurogentec, Fremont, CA, USA) on an ABI 7900HT quantitative Real-Time PCR machine. Results were analyzed with the standard delta cycle threshold method and were normalized to the transcription of Gapdh [38].

### 4.9. Flow Cytometry

Single-cell suspensions from muscle were prepared by collagenase II (Worthington Biochemical Corp, Lakewood, NJ, USA) digestion followed by staining with fluorochrome-conjugated antibodies.

Antibodies used: (1) Gr1 Clone RB6-8C5 (eBioscience, ThermoFisher, Waltham, MA, USA, Cat#48-5931-82); (2) F4/80 Clone BM8 (Invitrogen, Cat# 11-4801-82); (3) CD11b Clone M1/70 (Invitrogen, ThermoFisher, Waltham, MA, USA, Cat#17-0112-81); (4) Siglec-F Clone E50-2440 (RUO), (BD BioSciences, ThermoFisher, Waltham, MA, USA, Cat# 552126); (5) CD206 Clone C068C2 (BioLegend, San Diego, CA, USA, Cat#141701) and live dead stain (eBioscience). Data acquisition was performed on a FACS Aria or Fortessa (BD Biosciences). UltraComp eBeads (Cat#01333342, Invitrogen) were used to generate single-stain controls. Data was analyzed using FlowJo software (version 10.2) and gating strategies as previously described [39] to discriminate against dead cells, debris, and doublets were utilized. M1 macrophages were defined as Gr1^low-med^, F4/80^+^, and CD11b^+^.

### 4.10. Immune Response Challenge

*Salmonella enterica* serovar Typhimurium strain (χ3761) was inoculated into a 5 mL culture of Luria-Bertani (LB) broth (Cat# BP1426, ThermoFisher Scientific) and grown statically at 37 °C for 18 h. The overnight statically grown cultures were inoculated at a 1:50 dilution into fresh pre-warmed LB broth and grown with gentle aeration at 37 °C until OD_600_ = 0.85–0.9. Cultures were centrifuged at 6000× *g* for 15 min at room temperature and the pellet gently resuspended in 250 µL of sterile PBS. The culture was brought up to 500 µL with sterile PBS, serially diluted, and plated on LB agar to determine CFU per mL as the basis for determining the actual dose of the challenge strain. Mice were deprived of food and water for 6 h immediately prior to oral inoculation behind the incisors with 20 µL of the bacterial strain. Food and water were returned 30 min after oral inoculation. Five mice each were inoculated with 10^2^, 10^3^, 10^5^, and 10^7^ CFU *S. enterica* to determine the degree of virulence. LD_50_ values were calculated using the method of Reed and Muench [40].

### 4.11. Statistics

All experiments were performed using at least three biological replicates. Results were expressed as means ± SD. Statistical analysis was done using one-way ANOVA or Student’s t test performed in GraphPad and *p* ≤ 0.05 was considered significant (* *p* ≤ 0.05, ** *p* ≤ 0.01, *** *p* ≤ 0.001).

## 5. Conclusions

In this study, the complex role of *Mkx* was examined in acute muscle regeneration. *Mkx* is expressed in both the muscle and cells of the innate immune system. In examining muscle repair in mice deficient for *Mkx*, we found that the process was delayed and there was reduced recruitment of macrophages and eosinophils and secretion of cytokines and chemokines. These data indicate that *Mkx* has a complex role in the regulation of muscle regeneration.

## Data Availability

All data are contained in the paper.
